# Measuring health-related quality of life in Hungarian children with heart disease: psychometric properties of the Hungarian version of the Pediatric Quality of Life Inventory™ 4.0 Generic Core Scales and the Cardiac Module

**DOI:** 10.1186/1477-7525-8-14

**Published:** 2010-01-28

**Authors:** Andrea Berkes, István Pataki, Mariann Kiss, Csilla Kemény, László Kardos, James W Varni, Gábor Mogyorósy

**Affiliations:** 1 University of Debrecen Medical and Health Science Center, Department of Pediatrics, Nagyerdei krt. 98. Debrecen 4032, Hungary; 2 University of Debrecen Medical and Health Science Center, Department of Behavioral Sciences, Móricz Zsigmond krt. 22. Debrecen 4032, Hungary; 3 Kenézy Hospital, Hygiene and Infection Control Services, Bartók Béla út 2-26. Debrecen 4043, Hungary; 4 Department of Pediatrics, College of Medicine, Department of Landscape Architecture and Urban Planning, College of Architecture, Texas A&M University College Station, Texas, USA

## Abstract

**Objectives:**

The aim of the study was to investigate the psychometric properties of the Hungarian version of the Pediatric Quality of Life Inventory™ (PedsQL™) Generic Core Scales and Cardiac Module.

**Methods:**

The PedsQL™ 4.0 Generic Core Scales and the PedsQL™ 3.0 Cardiac Module was administered to 254 caregivers of children (aged 2-18 years) and to 195 children (aged 5-18 years) at a pediatric cardiology outpatient unit. A postal survey on a demographically group-matched sample of the general population with 525 caregivers of children (aged 2-18 years) and 373 children (aged 5-18 years) was conducted with the PedsQL™ 4.0 Generic Core Scale. Responses were described, compared over subgroups of subjects, and were used to assess practical utility, distributional coverage, construct validity, internal consistency, and inter-reporter agreement of the instrument.

**Results:**

The moderate scale-level mean percentage of missing item responses (range 1.8-2.3%) supported the feasibility of the Generic Core Scales for general Hungarian children. Minimal to moderate ceiling effects and no floor effects were found on the Generic Core Scales. We observed stronger ceiling than floor effects in the Cardiac Module. Most of the scales showed satisfactory reliability with Cronbach's α estimates exceeding 0.70. Generally, moderate to good agreement was found between self- and parent proxy-reports in the patient and in the comparison group (intraclass correlation coefficient range 0.52-0.77), but remarkably low agreement in the perceived physical appearance subscale in the age group 5-7 years (0.18) and for the treatment II scale (problems on taking heart medicine) scale of the Cardiac Module in children aged 8-12 years (0.39). Assessing the construct validity of the questionnaires, statistically significant difference was found between the patient group and the comparison group only in the Physical Functioning Scale scores (p = 0.003) of the child self-report component, and in Physical (p = 0.022), Emotional, (p = 0.017), Psychosocial Summary (p = 0.019) scores and in the total HRQoL (health-related quality of life) scale score (p = 0.034) for parent proxy-report.

**Conclusion:**

The findings generally support the feasibility, reliability and validity of the Hungarian translation of the PedsQL™ 4.0 Generic Core Scales and the PedsQL™ 3.0 Cardiac Module in Hungarian children with heart disease.

## Background

Medical progress has lead to increased survival, as a result a progressively increasing number of patients are living with congenital heart disease. This increased number of children living in that chronic condition means that clinicians face a multitude of challenges when caring for pediatric patients with congenital heart disease. The challenges are the impact of the condition on daily life and functioning, the psychosocial consequences, and the impact on individual psychological and social well-being [1]. There is an intense need for the opportunity to investigate and manage symptoms of "the new hidden morbidity" - problems of psychosocial health [2]. Focusing on the patients' psychological and social well-being in addition to their physical health is an essential requirement in accordance with the WHO definition of health and well-being [3]. Pediatric quality of life studies that investigate the individuals' perceptions of their well-being in a multidimensional aspect (physical and psychosocial dimensions) are a relatively new field of research in pediatric cardiology internationally, and meeting professional requirements in a pediatric population brings more difficulty than in adults: identifying the relevant quality-of-life components of these child-patients and how to measure them, showing sensitivity to the continuous and rapid cognitive and emotional development of children, getting information from the patient and from a parent simultaneously, dealing with response-shift, in addition to the general requirements such as ensuring comparability of populations living in different conditions by using instruments with generic cores and disease specific modules, and adaptation of questionnaires to several languages and cultures [4-11]. Recent literature gives us an increasing volume of evidence that these studies can have an important role in the care of chronically ill children [12-22].

Results of a large sample study assessing health-related quality of life with a multidimensional, well-validated and reliable instrument reveal that approximately 20% of children with heart disease report significantly impaired psychosocial quality of life irrespective of the severity of heart disease [23]. This recent result affirms previous findings of studies focusing on various dimensions of quality of life [21,24-27].

As congenital heart diseases in Hungary, like elsewhere, are the most frequent group of congenital abnormalities with very good biological prognosis, and because the incidence of psychosocial problems is even greater in the Hungarian general population than in other European countries [28,29] we considered it necessary to assess the health-related quality of life of Hungarian children with heart disease.

Among several instruments we decided to use the Pediatric Quality of Life Inventory™, which is a modular instrument with numerous disease specific modules, already utilized in many translated versions, and with forms available for a wide range of ages (2-18 years) [30-35]. The validity and reliability of the instrument has been confirmed as a population health measurement tool and in different child populations with chronic illnesses in descriptive and evaluative studies [9,16,17,20,22,36-38].

The PedsQL™ 4.0 Generic Core Scales differentiated health-related quality of life of healthy children and children with a chronic condition with good efficacy, and apart from the Generic Core Scales, in a large sample study on children with congenital heart disease, the severity of cardiac disorder was also reflected by the Cardiac Module [23,36,39].

The current study presents the psychometric properties of the Hungarian version of the PedsQL™ 4.0 Generic Core Scales and the PedsQL™ 3.0 Cardiac Module estimated on samples from the general Hungarian child population and from children with heart diseases.

## Methods

### Participants and settings

Potential study subjects were recruited from the Pediatric Cardiology Outpatient Unit of the University of Debrecen Medical and Health Science Centre, Department of Pediatrics. Subjects of the comparison group were chosen by random selection from the general Hungarian population through the Population Register Office of the Ministry of the Interior, with distributional matching to the population treated at the pediatric cardiology outpatient unit on age, gender, and residence.

Subjects were given detailed written information about the methods, aims, and the voluntary nature of participation in the study. Subjects of the patient group filled in the questionnaires in a room inside the outpatient clinic, while data collection from the comparison group was carried out through mail correspondence. Subjects of the patient group were excluded from participation if the child had associated non-cardiac chronic disease or major developmental disability, mental retardation that might affect health-related quality of life, and if the child was < 2 months after surgical intervention. 38 children were excluded because the child had associated non-cardiac chronic disease or major developmental disability, severe mental retardation. The most frequent disorders were hematologic diseases, asthma bronchiale, diabetes mellitus, epilepsy, which were not results of any kind of heart diseases. Mild somatomental retardation, which was observable in some children with CHD of great complexity, could be a consequence of the heart disease, but these children were not excluded from the study. No children were excluded due to psychological problems. All the diagnoses of usual occurrence at a pediatric outpatient unit were represented in the patient sample. Patients with congenital heart disease were classified according to the guidelines set at the 32nd Bethesda Conference of the American College of Cardiology [40], and they were categorized into three groups, namely simple congenital heart disease (such as isolated small or repaired atrial and septal defect without residua), congenital heart disease with moderate complexity (for example, coarctation of the aorta, moderate-to-severe pulmonary valvar disease or tetralogy of Fallot), and great complexity (such as double-outlet ventricle or conditions with conduits or after Fontan procedure). Beside congenital heart defects the study sample included patients with cardiomyopathies, arrhythmias and acquired (such as carditis, Kawasaki syndrome) heart diseases. The research protocol was approved by the Research Ethics Committee of The University of Debrecen.

### Measures

The PedsQL™ Measurement Model is a modular approach to measure HRQoL for a wide age range of children and adolescents from 2 to 18 years of age. The development, refinement and validation of the original instrument and linguistic validation to a number of European and other languages have been described in many papers [30-35]. Results of research with disease-specific modules are available [13,14,16,17,41]. Methodology of application and evaluation can be found in several previous presentations [9,42].

The 23-item PedsQL™ 4.0 Generic Core Scales encompass: 1) Physical Functioning (8 items), 2) Emotional Functioning (5 items), 3) Social Functioning (5 items), and 4) School Functioning (5 items), and were developed through focus groups, cognitive interviews, pre-testing, and field testing measurement development protocols. Cognitive interviews were carried out with children attending the pediatric cardiology outpatient unit. Five children were chosen from each age group, with different severities of heart disease, from different places of residence. To get information on children without proven heart disease, interviews were performed with 4 children with innocent heart murmur.

The PedsQL™ 4.0 Generic Core Scales are comprised of parallel child self-report and parent proxy-report formats. Child self-report includes ages 5-7, 8-12, and 13-18 years. Parent proxy-report includes ages 2-4 (toddler), 5-7 (young child), 8-12 (child), and 13-18 (adolescent), and assesses parent's perceptions of their child's HRQOL. The items for each of the forms are essentially identical, differing in developmentally appropriate language, or first or third person tense. The instructions ask how much of a problem each item has been during the past one month. A 5-point response scale is utilized across child self-report for ages 8-18 and parent proxy-report (0 = never a problem; 1 = almost never a problem; 2 = sometimes a problem; 3 = often a problem; 4 = almost always a problem). To further increase the ease of use for the young child self-report (ages 5-7), the response scale is reworded and simplified to a 3-point scale (0 = not at all a problem; 2 = sometimes a problem; 4 = a lot of a problem), with each response choice anchored to a happy to sad faces scale. Parent proxy-report also includes the toddler age range (ages 2-4), which does not include a self-report form given developmental limitations on self-report for children younger than 5 years of age, and includes only 3 items for the school functioning scale.

Items are reverse-scored and linearly transformed to a 0-100 scale (0 = 100, 1 = 75, 2 = 50, 3 = 25, 4 = 0), so that higher scores indicate better HRQOL. Scale Scores are computed as the sum of the items divided by the number of items answered (this accounts for missing data). If more than 50% of the items in the scale are missing, the Scale Score is not computed [9,42]. In addition to the single scale scores there is the possibility to calculate summary scores: the Physical Health Summary Score is the same as the Physical Functioning Subscale, whereas to create the Psychosocial Health Summary Score, the mean is computed as the sum of the items divided by the number of items answered in the Emotional, Social, and School Functioning Subscales.

The sequential validation procedure of the original U.S. version of the PedsQL™ 3.0 Cardiac Module was carried out by instruction of the MAPI Research Institute, in accordance with the guidelines of the QOL-SIG TCA (Quality of Life - Special Interest Group Translation and Cultural Adaptation) group [43-47].

The PedsQL™ 3.0 Cardiac Module was translated independently into Hungarian by two professional translators, native target language speakers, bilingual in the source language. The two translated versions of the questionnaires were discussed with both translators, a pediatric cardiologist, a pediatrician, a nurse in pediatric cardiology, and a teacher, and the final combined version was back translated into English. After review and comments by the instrument author, the new version was tested on 20 parents of children with heart disease aged 2-18 years and 15 children aged 5-18 years by cognitive interviews. These interviews were performed to determine whether any questions were difficult to understand and/or irrelevant. After some modification on wording and proofreading, the final version was forwarded to the MAPI Research Institute, which gave the approval for the psychometric probe of the Hungarian PedsQL™ 3.0 Cardiac Module. The format, instructions, Likert response scale, and scoring method for the PedsQL™ 3.0 Cardiac Module are identical to the PedsQL™ 4.0 Generic Core Scales, with higher scores indicating better HRQOL (fewer symptoms or problems).

Our study group took part in the adaptation process for the PedsQL™ 3.0 Cardiac Module only; the Hungarian Generic Core Scale was already available through the MAPI Research Institute.

### Statistical analysis

*Feasibility *of the Hungarian version of the Cardiac Module was determined from the average percentage of missing responses. The percentage of all possible item-responses left unanswered was calculated for each subject on each single and summary scale and averaged over subjects. The utility of the instruments in terms of distributional coverage overall and by subscale was evaluated by calculating the percentage of subscale-level average responses reaching the minimum (floor) or the maximum (ceiling) of the scoring scale.

*Construct validity *was determined using the known groups method. PedsQL™ Generic Core Scales scores were compared between groups differing in known health conditions. HRQoL scores of children from the general population and children with heart diseases were compared using t tests for independent samples. Effect sizes were evaluated using Cohen's d statistics [48]. Construct validity of the Cardiac Module was further assessed by estimating the intercorrelations among the Cardiac Module scale scores and relevant Generic Core Scales scores [48].

Scale *internal consistency *reliability was determined by calculating Cronbach's coefficient α. *Agreement between self-report and parent proxy-report *was assessed using the Pearson correlation coefficient (with thresholds for medium and large correlation at 0.30 and 0.50, respectively), the intraclass correlation coefficient for absolute agreement (ICC, interpreted using thresholds for moderate and good agreement at 0.4 and 0.6, respectively) [49], Bland-Altman 95% limits of agreement (LOA) [50], and by evaluating parent vs. child mean score differences in paired *t *tests.

## Results

### Sample characteristics

The Hungarian translations of the PedsQL™ 4.0 Generic Core Scales and the PedsQL™ 3.0 Cardiac Module were administered to 195 children attending the cardiology outpatient unit aged 5-18 years and 254 parents of children aged 2-18 years. It was the mother who answered the questionnaire in 92.52% of the sample, and it was the father in 7.48% of the sample. No parent in the patient group refused to participate in the study, 3 patients ages 5-7 years were unwilling to answer during the interview.

Of 1000 families approached by mail, 525 families as subjects of the comparison group were recruited into the study (52.5%). Subjects included 268 boys (51.05%) and 215 girls (40.95%) and 42 (8%) of unknown gender. It was the mother who answered the questionnaire in 89.5% of the sample, it was the father in 4.57% of the sample, and it was someone else in 6.28% of the sample. Distribution of all participants in terms of gender and age group is shown in Table 1.

**Table 1:** Sample characteristics

Scale	Total sample		Male		Female		Unknown gender	
	
	Number	Percent	Number	Percent*	Number	Percent*	Number	Percent**
Patient group								
Toddler (2-4)	59	23.23	34	57.63	25	42.37		
Young child (5-7)	49	19.29	27	55.10	22	44.90		
Child (8-12)	73	28.74	43	58.90	30	41.10		
Adolescent (13-18)	73	28.74	44	60.27	29	39.73		
All ages	254	100.00	148	58.27	106	41.73		
Comparison group								
Toddler (2-4)	152	28.95	81	56.25	63	43.75	8	5.26
Young child (5-7)	111	21.14	58	58.59	41	41.41	12	10.81
Child (8-12)	160	30.48	72	50.00	72	50.00	16	10.00
Adolescent (13-18)	102	19.43	57	59.38	39	40.63	6	5.88
All ages	525	100.00	268	55.49	215	44.51	42	8.00

*Row percentages with known-gender subjects taken as 100%

**Percentages with Number under total sample taken as 100%

### Feasibility

Missing values were found for the patient group's Generic Core Scale (ranging 13.8-25.9%), with highest values in the school functioning domain both for both self- and parent proxy-reports, and in the Cardiac Module (ranging 0.5-66.2%) with highest values in the Treatment II Scale (problems with taking heart medicine) domain. The percentages of missing values (ranging 1.2 - 4.4%) in the comparison group were consistent with previous results (Tables 2, 3).

**Table 2:** Scale descriptives, average missing item percentages skewness and Cohen's d values for the Pediatric Quality of Life Inventory™ 4.0 Generic Core Scales child self-report (195 patient and 373 comparison group subjects) and parent proxy-report (254 patient and 525 comparison group subjects), comparing the patient and comparison groups

	Patient group						Comparison group						
**Scale**	**N**	**Mean**	**S.D.**	**Missing values (%)**	**Percent floor (%)**	**Percent ceiling (%)**	**N**	**Mean**	**S.D.**	**Missing values (%)**	**Percent floor (%)**	**Percent ceiling (%)**	**Cohen's d**

Child Self-report													
Total Scale Score	164	76.86	14.64	14.30	0.00	0.00	366	79.33	12.35	2.00	0.00	2.50	0.19
Physical functioning	164	78.26**	18.81	13.90	0.00	11.00	366	83.12	14.23	2.00	0.00	13.70	0.31
Psychosocial functioning	164	76.09	14.47	14.50	0.00	3.00	366	77.29	13.39	2.10	0.00	3.00	0.09
Emotional functioning	164	71.71	17.07	13.80	0.00	6.70	365	72.1	17.80	2.00	0.00	8.20	0.02
Social functioning	164	82.59	17.54	13.90	0.00	28.00	366	83.81	16.10	1.80	0.30	28.70	0.07
School functioning	160	73.94	16.82	15.80	0.00	7.50	364	75.84	16.65	2.30	0.00	10.70	0.11
Parent Proxy-report													
Total Scale Score	212	76.02*	15.3	17.00	0.00	0.90	519	78.85	13.18	1.80	0.20	2.10	0.20
Physical functioning	212	77.66*	18.73	15.30	0.00	14.60	519	81.03	15.88	1.30	0.20	13.10	0.20
Psychosocial functioning	212	75.06*	15.49	18.00	0.00	1.90	519	77.66	13.69	2.10	0.20	2.70	0.18
Emotional functioning	212	68.45*	18.06	15.00	0.00	5.20	519	71.79	16.76	1.20	0.20	7.50	0.20
Social functioning	212	82.13	19.68	15.30	0.00	30.20	518	84.45	16.31	1.50	0.20	31.70	0.13
School functioning	183	74.55	18.62	25.90	0.00	11.50	502	77.01	16.93	4.40	0.00	13.70	0.14

N = Number of valid cases; S.D. = Standard deviation; *Difference between cardiac and healthy samples significant at p < 0.05; **Difference between cardiac and healthy samples significant at p < 0.005; missing value percentages are averaged over all subjects

**Table 3:** Scale descriptives, average missing item percentages and skewness for the Pediatric Quality of Life Inventory™ 3.0 Cardiac Module child self-report (195 subjects) and parent proxy-report (254 subjects)

Cardiac module	N	Mean	S.D.	Missing values (%)	%Floor	%Ceiling
Child Self-report						
Total Scale Score	187	77.68	13.50	15.00	0.00	8.90
Heart problems-symptoms	191	76.42	17.08	0.50	0.00	66.30
Treatment II	83	93.19	13.09	66.20	0.00	39.50
Perceived physical appearance	172	83.14	19.45	9.90	1.60	37.20
Treatment anxiety	188	78.29	25.27	4.00	0.00	11.20
Cognitive problems	178	73.04	19.44	7.70	2.60	29.10
Communication	189	74.25	26.08	1.60	0.00	1.10
Parent Proxy-report						
Total Scale Score	251	76.19	14.62	14.60	0.00	7.90
Heart problems-symptoms	252	76.40	17.46	0.70	0.00	77.90
Treatment II	95	93.73	15.75	65.80	0.40	47.50
Appearance	223	82.83	23.00	11.60	1.20	24.80
Treatment anxiety	250	69.77	26.73	1.90	0.00	19.00
Cognitive problems	237	73.41	21.03	8.60	3.70	35.70
Communication	241	74.50	28.31	1.10	0.00	2.00

N = Number of valid cases; S.D. = Standard deviation; missing value percentages are averaged over all subjects

### Descriptive statistics

As evident from Table 2, no floor effects were seen on the Generic Core Scales. We found ceiling effects both in child self- and parent proxy-reports ranging from a minimal 0.9 to a moderate 30.2% in the patient group and 2.1-31.7% in the comparison group, with highest values in the Social Functioning Scale for child self- and parent proxy-reports from the patient and comparison samples. We also observed greater ceiling (1.1-77. 9%) than floor effects (0.4-3.7%) in the Cardiac Module, with a notable ceiling effect in the Heart Symptoms scale and a moderate one in the Treatment II Scale, Perceived Physical Appearance, and Cognitive Problems Scales subscales for child self- and parent proxy-reports (Table 3.). Cronbach's coefficient α estimates for the PedsQL™ Generic Core Scales and for the Cardiac Module across all ages of the patient and comparison groups are presented in Tables 4. and Table 5. The recommended standard of 0.70 for group comparison was exceeded in the majority of the scales, and all scales exceeded the satisfactory level of internal consistency reliability of at least 0.40.

**Table 4:** Internal consistency reliability for Pediatric Quality of Life Inventory™ 4.0 Generic Core Scales child self-report and parent proxy-report

Scale	Total sample		Toddler (2-4)		Young child (5-7)		Child (8-12)		Adolescent (13-18)	
	
	Patient group	Comparison group	Patient group	Comparison group	Patient group	Comparison group	Patient group	Comparison group	Patient group	Comparison group
	
	Cronbach's α									
Child Self-report										
Total scale score	0.90	0.87			0.83	0.78	0.92	0.91	0.90	0.88
Physical functioning	0.82	0.75			0.67	0.62	0.89	0.80	0.79	0.80
Psychosocial functioning	0.86	0.82			0.80	0.72	0.87	0.89	0.87	0.84
Emotional functioning	0.69	0.71			0.55	0.61	0.71	0.77	0.77	0.73
Social functioning	0.75	0.72			0.60	0.48	0.75	0.79	0.78	0.83
School functioning	0.68	0.68			0.59	0.51	0.66	0.78	0.74	0.74
Parent Proxy-report										
Total scale score	0.91	0.89	0.90	0.91	0.90	0.89	0.92	0.88	0.90	0.88
Physical functioning	0.84	0.82	0.86	0.87	0.76	0.78	0.87	0.80	0.82	0.82
Psychosocial functioning	0.88	0.84	0.83	0.84	0.87	0.84	0.88	0.85	0.88	0.84
Emotional functioning	0.77	0.73	0.73	0.75	0.74	0.7	0.80	0.74	0.81	0.74
Social functioning	0.83	0.76	0.80	0.78	0.88	0.78	0.79	0.70	0.85	0.80
School functioning	0.75	0.71	0.59	0.43	0.74	0.70	0.79	0.74	0.70	0.75

**Table 5:** Internal consistency reliability for Pediatric Quality of Life Inventory™ 3.0 Cardiac Module child self-report and parent proxy-report

Scale	Total patient group	Toddler (2-4)	Young child (5-7)	Child (8-12)	Adolescent (13-18)
	
	Cronbach's α				
Child Self-report					
Total score	0.87		0.65	0.90	0.89
Heart problems-symptoms	0.75		0.58	0.77	0.81
Treatment II	0.64		0.50	0.56	0.73
Appearance	0.65		0.58	0.65	0.67
Treatment anxiety	0.89		0.92	0.87	0.89
Cognitive problems	0.72		0.60	0.76	0.78
Communication	0.76		0.75	0.74	0.83
Parent proxy-report					
Total score	0.89	0.70	0.70	0.89	0.91
Heart problems-symptoms	0.80	0.80	0.79	0.78	0.83
Treatment II	0.82	0.84	0.85	0.71	0.86
Appearance	0.73	0.54	0.49	0.73	0.72
Treatment anxiety	0.89	0.92	0.84	0.88	0.91
Cognitive problems	0.80	0.78	0.63	0.78	0.80
Communication	0.86	0.96	0.78	0.80	0.87

### Construct validity

Assessing the construct validity of the questionnaires, statistically significant difference was found between the patient group and the comparison group in just Physical Functioning Scale (p = 0.003) scores of the child self-report for the Generic Core Scales. For parent proxy-reports, statistically significant difference was found in the Physical Functioning Scale (p = 0.022), Emotional Functioning Scale (p = 0.017), and Psychosocial summary score (p = 0.019), and also in the Total Scale Score (p = 0.034) (Table 2). Mean scores were consistently higher in the comparison group for all scales, with Cohen's d values indicating no other than small effects (range 0.02-0.31).

As to the intercorrelations among the various Generic Core Scales and the Cardiac Module scales estimated using Pearson correlation coefficients, a high correlation was found between the Physical Functioning Scale scores and Cardiac Symptoms Scale scores for children (r = 0.63) and for parents (r = 0.66). Cognitive Problems Scale scores of the Cardiac Module were highly correlated with the School Functioning Scale (self-reports r = 0.57, proxy-reports r = 0.60), the Psychosocial Summary scores (both reports r = 0.58), and with the Total Scale Score (self-reports r = 0.58, proxy-reports r = 0.58) of the Generic Core Scale (Table 6).

**Table 6:** Intercorrelations of subscales of the Pediatric Quality of Life Inventory™ Generic Core Scales and Cardiac Module assessed with Pearson correlation coefficient

	Cardiac module					
	
	Heart-problems-symptoms	Treatment II	Perc. Phys. appearance	Treatment anxiety	Cognitive problems	Communication
Generic core scales						
Child Self-report						
Total	0.544	0.27	0.45	0.39	0.58	0.46
Physical functioning	0.63	0.29	0.35	0.34	0.46	0.401
Psychosocial functioning	0.41	0.23	0.45	0.37	0.58	0.44
Emotional functioning	0.38	0.24	0.43	0.38	0.47	0.38
Social functioning	0.35	0.13	0.38	0.27	0.45	0.41
School functioning	0.32	0.27	0.35	0.30	0.57	0.34
Parent Proxy-report						
Total	0.57	0.47	0.40	0.35	0.57	0.45
Physical functioning	0.66	0.33	0.34	0.29	0.43	0.36
Psychosocial functioning	0.43	0.49	0.38	0.34	0.58	0.45
Emotional functioning	0.33	0.45	0.39	0.37	0.41	0.38
Social functioning	0.32	0.37	0.28	0.24	0.44	0.37
School functioning	0.41	0.43	0.26	0.22	0.60	0.37

Effect sizes are designated as small (0.10), medium (0.30) and large (0.50)

### Parent-child agreement

Table 7 presents the ICCs between child self-reports and parent proxy-reports of the PedsQL™ 4.0 Generic Core Scales and the PedsQL™ 3.0 Cardiac Module. Moderate to good agreement was found in the Generic Core Scales of both the patient and comparison groups. ICCs were generally higher in the comparison group. Lower values were obtained in the Emotional and Social Functioning Scales across all age groups, and in the School Functioning Scale in 5-7 and 13-18 year-olds from the patient group. All ICCs showed good agreement in the comparison group, except for the Physical and Social Functioning Scale scores of children aged 5-7 years. ICCs for the Cardiac Module indicated similarly moderate to good agreement, with lower values for the Treatment II Scale, Perceived Physical Appearance Scale, and the Treatment Anxiety Scale in most age groups. Poor agreement was detected in the Perceived Physical Appearance Scale for the 5-7 year olds and in the Treatment II Scale for the 8-12 year olds. The ranges of LOA as calculated following the Bland-Altman procedure are consistent with the mainly moderate agreements between child self- and proxy-report scales. Neither the ICC nor the LOA values indicate any tendency of improvement in parent-child agreement as age advances (data for LOA by age group not shown).

**Table 7:** Agreement between self-report and parent proxy-report Pediatric Quality of Life Inventory™ 4.0 Generic Core Scales and for the Pediatric Quality of Life Inventory™ 3.0 Cardiac Module scales

Scale	Intraclass correlation coefficients				Difference		
	
	5-7 year-olds	8-12 year-olds	13-18 year-olds	All ages	Mean	P	LOA
Generic Core Scale Patient group							
Total	0.68	0.78	0.62	0.71	-1.28	0.161	-20.86; 23.42
Physical functioning	0.60	0.81	0.66	0.72	-1.01	0.360	-25.76; 27.77
Psychosocial functioning	0.63	0.69	0.61	0.65	-1.45	0.152	-23.07; 25.96
Emotional functioning	0.47	0.56	0.50	0.52	-3.28	*0.020*	-30.75; 37.31
Social functioning	0.52	0.48	0.66	0.57	-0.86	0.529	-32.57; 34.30
School functioning	0.33	0.71	0.55	0.57	-0.48	0.722	-31.47; 32.43
Generic Core Scale Comparison group							
Total	0.73	0.75	0.75	0.74	-1.01	0.052	-16.86; 18.87
Physical functioning	0.53	0.63	0.74	0.64	-2.47	*0.001*	-22.72; 27.66
Psychosocial functioning	0.75	0.78	0.73	0.76	-0.23	0.670	-18.25; 18.70
Emotional functioning	0.63	0.75	0.71	0.70	-1.14	0.146	-25.83; 28.10
Social functioning	0.54	0.73	0.63	0.66	0.62	0.416	-26.83; 25.60
School functioning	0.67	0.77	0.74	0.73	-0.08	0.906	-24.34; 24.51
Cardiac Module Patient group							
Heart problems-symptoms	0.73	0.84	0.71	0.77	-1.29	0.145	-21.57; 24.15
Treatment II.	0.47	0.39	0.65	0.54	-0.35	0.875	-32.44; 33.14
Appearance	0.18	0.55	0.58	0.53	-5.13	*0.004*	-36.83; 47.08
Treatment anxiety	0.59	0.46	0.61	0.55	-8.09	*0.000*	-39.01; 55.20
Cognitive problems	0.67	0.61	0.68	0.65	-2.88	*0.028*	-29.45; 35.21
Communication	0.70	0.69	0.54	0.64	-0.85	0.626	-44.23; 45.94

Negative signs in mean difference indicate proxy-report scores being lower; LOA = Bland-Altman 95% Limits of Agreement

## Discussion

This article describes the psychometric properties of the Hungarian version of the PedsQL™ 4.0 Generic Core Scale and the PedsQL™ 3.0 Cardiac Module.

The findings generally support the feasibility, reliability and validity of the Hungarian translations of the generic core and cardiac-specific instruments to assess HRQoL of Hungarian children 2-18 years of age.

The marked difference in missing values between the patient and the comparison group highlight the importance of situational circumstances at the time of the survey. In a medical institution, potential subjects tend to agree to participate much more willingly when asked by medical staff. On the other hand, patient and parent stress and time limitations could be factors that explain incompleteness of filling-in the questionnaire. In the postal survey of the comparison group, respondents' willingness was not influenced by any extraneous factors such as illness, fatigue and time limitations. Further, the general population was requested to only complete the Generic Core Scales, while the cardiac sample was additionally requested to complete the Cardiac Module, which may increase respondent burden.

For the Cardiac Module, extremely high frequencies of missing values were detected for the Treatment II Scale (taking heart medication) and in the Perceived Physical Appearance subscales. Although there is an instruction in the questionnaire to skip the Treatment II Scale if the child does not take heart medication, many respondents failed to take notice of it this instruction. A written or - when it is possible - verbal notice might induce more focused attention and decrease the bias due to missing values. By deleting the missing values from the Treatment II Scale from the calculations, missing value percentages for the total cardiac module decrease from 15.0% to 5.4% for child self-report, and from 14.6% to 4.8% for parent proxy-report. The high proportion of patients without surgical treatment could result in a similar augmentation for the Perceived Physical Appearance Scale. As Hungarian children under 7 do not attend school, and because the social support system allows schooling to be postponed for children with chronic conditions, an over-representation of pre-school respondents may have raised the missing value frequencies for the Cognitive Functioning Scale. Other European investigators also reported that the daycare or school functioning subscale is not applicable for children aged 2-7 years [11,30].

The PedsQL™ 4.0 Generic Core Scales indicated better HRQoL in children of the general population than in children with heart disease consistently on all scales, which supported the construct validity of the translated instrument. The impaired physical functioning of children with more severe heart diseases has already been demonstrated by the PedsQL™ [23] but was not observable on a smaller sample with different severities of heart disease [17]. This finding could reflect the lack of physical activities and their serious restrictedness [26]. Although heart diseases from a medical point of view have influence primarily on physical states, the majority of HRQoL studies found expressed deficits in psychosocial dimensions [17,23,51-53]. Concordantly with these previous findings, our data on parent proxy-reports also showed significant differences in the Emotional Functioning Scale and the Psychosocial Summary Score, and in the Total Generic Core Scales Score. This observation may indicate the parental underestimation of certain dimensions of HRQoL and the advanced levels of children's coping strategies [4,54-57]. Subscale values were highest in the Social Functioning Scale, probably indicating the successful integration of children with heart disease into their peer group [25]. The low scores on the Emotional Functioning Scale suggest the children's distress associated with their chronic condition [21,55,58-60]. The sample consisted of children with different severity of heart disease. The ratio of children with severe to those with simple heart diseases corresponded to the distribution of patients attending a typical pediatric cardiology outpatient unit. According to our and to previous results, quality of life of children with different severity of heart diseases - as a whole group - does not differ significantly from that of the general population [17]. It means that the justification for stigmatization of heart disease, with its negative consequences, is strongly refuted by the children themselves. Thanks to the enormous advance in pediatric cardiac surgery, most congenital heart diseases can be resolved by interventions, ensuring good quality of life for children.

Intercorrelations estimated by this study between generic core scales and cardiac module scales are consistent with the previous literature [17].

No (for Generic Core Scales) or minimal (for the Cardiac Module) floor effects and more accentuated ceiling effects for both scales means that distinction by the Hungarian translation of the instrument between persons who do extremely well or just well is less than excellent [14,30,61-63]. Child and parent scores from the comparison group showed stronger ceiling effects than those from the patient group, as would be expected. Highest values appearing on the Social Functioning Scale can also be a sign of the success of coping mechanisms or peer acceptance. The notable ceiling effect in the heart symptoms subscale of the Cardiac Module is understandable in a mixed population of children with different heart disease severity, where a considerable proportion of the sample do not have a severe condition which would be expected to influence markedly their daily lives. Moderate ceiling effects in the Treatment II, Perceived Physical Appearance, and Cognitive Problems Scales for child self- and parent proxy-report are also consistent with the diversity of disease severity of the studied population, with some patients not taking heart medicine and having had no cardiac intervention.

Consistently with previous findings, some lower internal consistency reliability values were calculated in younger age groups [9,64] and for the Social and School Functioning Scales of the Generic Core Scales and for the Treatment II, Perceived Physical Appearance, and Cognitive Functioning Scales of the Cardiac Module, where small sample size could possibly compromise the precision of results.

Regarding the agreement between child self- and parent proxy-reports, our data showed generally moderate to good agreement both for the Generic Core Scales and the Cardiac Module. Finding higher correlations for the observable parameters in general, like the Physical Functioning Scale in the Generic Core Scale and heart symptoms, communication and cognitive functioning in the Cardiac Module is consistent with previous literature [7,17,30]. In the patient group, lower agreement was observable on school functioning in children aged 5-7 and 13-18 years. The low representation of schoolchildren among chronically ill children between 5-7 years may have biased these results. The particularly low agreement on the Perceived Physical Appearance Scale of the Cardiac Module in the age group 5-7 years could indicate unrecognized anxiety. Perceptions of being different from the others, the possible peer discrimination because of the presence of a scar on the chest in the usual period of starting to go to kindergarten or school may cause hidden distress. Another ICC value indicating poor agreement was found for the Treatment II Scale in the 8-12 age group. It is commonly known that compliance to taking medicine in the period of early adolescence is declining but may remain unrecognized by the parents [65-67]. Our data do not confirm the findings of higher parent-child agreement among chronically ill children as the majority of ICCs were higher in the comparison group [7]. We did not investigate other factors (like children's age, emotional state, parent's HRQoL, statistical method) that could also influence parent-child agreement [4,10,11]. Our findings confirm the need for the parallel application of child self- and parent proxy-reports in pediatric research [11,17,68]. The parental underestimation of QOL and coping mechanisms of chronically ill children is known from the literature [4,11,21,25]. The psychosocial support of the family should be the part of health care of chronically ill children. In light of the apparent limitations of parents' assessments in approximating children's true QoL, judgment must rely strongly on children's independent responses, which essentially requires instruments that are formulated in a child-friendly way.

Certain limitations exist in the study. Although the method of selecting subjects of the comparison group was designed to achieve a control set comparable to the patient group in terms of age and gender composition, the response rate - even though not differing significantly from other larger postal studies - was not sufficient to accomplish optimal demographic matching of the two groups. We also do not have sociodemographic information on the non-participants of the comparison group.

The situational context of questionnaire completion at the clinic or at home also needs consideration. The influence of site of administration on response rates has not been widely investigated, although mode of administration (in person versus mail survey) has been widely studied. A related issue is the incompleteness of answers from those who do respond. This limitation manifested strongly on one particular scale and can be improved upon as detailed above.

Another limitation of the study is that it does not report data across cardiac disease stages. The differences between children with severe cardiac disease and the general population would be probably larger [23]. The timing of inclusion may also have a great impact on HRQoL studies of patients with chronic conditions [69]. Pediatric subjects with congenital heart diseases could have been operated on at various lengths of time before being surveyed, but they were at least 2 months after the intervention. This important additional factor influencing HRQoL is not taken into account in our study, and should be studied systematically in future investigation of pediatric patients with cardiac conditions. Finally, this study does not provide data on test-retest reliability, which should be an additional goal of future investigations.

## Conclusion

Our results generally support the feasibility, reliability and validity of the Hungarian translation of PedsQL™ 4.0 Generic Core Scales and the PedsQL™ 3.0 Cardiac Module, but highlight the importance of situational settings during completion and the necessity of explicit instructions for several scales. Although the data from our study presents reasonable evidence for the psychometric properties of the Hungarian translation of the PedsQL™ 4.0 Generic Core Scales and PedsQL™ 3.0 Cardiac module for HRQoL studies in Hungarian children, future investigation with the instrument on larger samples of healthy children and on children with various levels of heart disease severity are recommended. Research focus should extend to other clinical populations, also testing sensitivity and responsiveness in longitudinal studies. The Hungarian translation of the PedsQL™ may further facilitate international comparisons and analysis of pediatric health care outcomes across countries [70].

## Competing interests

Dr. Varni holds the copyright and the trademark for the PedsQL™ and receives financial compensation from the Mapi Research Trust, which is a nonprofit research institute that charges distribution fees to for-profit companies that use the Pediatric Quality of Life Inventory™. The PedsQL™ is available at the PedsQL™ website [71].

## Authors' contributions

AB, CsK and GM designed the study. IP and MK collected the data. LK performed the statistical analyses. AB drafted the manuscript and participated in the statistical analyses. JWV and GM revised the manuscript critically. All authors read and approved the final manuscript.
